# Local adaptation to the native environment affects pyrethrin variability in Dalmatian pyrethrum populations

**DOI:** 10.3389/fpls.2024.1404614

**Published:** 2024-06-21

**Authors:** Martina Grdiša, Nina Jeran, Filip Varga, Zlatko Liber, Ante Turudić, Zlatko Šatović

**Affiliations:** ^1^ Department of Plant Biodiversity, Faculty of Agriculture, University of Zagreb, Zagreb, Croatia; ^2^ Centre of Excellence for Biodiversity and Molecular Plant Breeding (CoE CroP-BioDiv), Zagreb, Croatia; ^3^ Department of Biology, Faculty of Science, University of Zagreb, Zagreb, Croatia

**Keywords:** bioclimatic factors, Mediterranean, natural insecticides, specialized metabolites, *Tanacetum cineraiifolium*

## Abstract

The insecticidal compound pyrethrin is synthesized in Dalmatian pyrethrum (*Tanacetum cinerariifolium* (Trevis.) Sch.Bip.; Asteraceae), a plant species endemic to the eastern Mediterranean. Pyrethrin is a mixture of six compounds, pyrethrin I and II, cinerin I and II, and jasmolin I and II. For this study we sampled 15 natural Dalmatian pyrethrum populations covering the entire natural distribution range of the species; Croatian coastal regions and the islands, inland Bosnia and Herzegovina and Montenegro. The plants were grown in a field experiment under uniform growing conditions to exclude a short-term response to environmental factors and instead observe variation in pyrethrin content and composition among and within populations due to genetic adaptation to the native environment. The drivers of local adaptation were explored by examining the role of bioclimatic factors as a cause of population differentiation. Pyrethrins were extracted by ultrasound-assisted extraction, and the extracts were analyzed by HPLC-UV-DAD. The populations differed significantly in the content and composition of pyrethrins. The highest levels of total pyrethrins (1.27% flower DW), were found in population P14 Budva and the significantly highest levels of pyrethrin I in population P14 Vranjske Njive, Podgorica (66.47% of total pyrethrin). Based on bioclimatic conditions of the sampling sites, populations were grouped into five bioclimatic groups (A, B, C, D, and E), which showed qualitative and quantitative variability in pyrethrin content. The most abundant bioclimatic group was bioclimatic group E, which was characterized by the highest average values for pyrethrin I (53.87% of total pyrethrin), total pyrethrin content (1.06% flower DW) and the ratio of pyrethrin I and II (1.85). The correlation analysis between the pyrethrin compounds and some of the bioclimatic variables (e. g., BIO03 Isothermality and BIO04 Temperature seasonality) showed their significant contribution in explaining the variation of pyrethrins in *T. cinerariifolium*. The differences in pyrethrin content and composition may be partly due to genetic adaptation to the ecological conditions of the native environment. The obtained data would enable the selection of source populations for breeding programs aimed at producing cultivars with desirable biochemical properties and adaptation to different bioclimatic conditions.

## 1 Introduction

In general, the role of specialized metabolites in plants is diverse, ranging from defense against pathogens and herbivores to attracting pollinators and/or dispersal, protection against environmental stress, etc (Isah, 2019; Huang and Dudareva, 2023). Variations in the content of specialized metabolites in individual plant species depend on various factors (Pavarini et al., 2012; Pant et al., 2021), including abiotic components of the environment (Moore et al., 2014; Yang et al., 2018). They play an essential role in the interaction of plants with the environment and their ability to tolerate abiotic stress (Ashraf et al., 2018). Individuals of the same plant species that thrive under different ecological conditions may differ markedly in the synthesis and accumulation of specialized metabolites (Theis and Lerdau, 2003; Ramakrishna and Ravishankar, 2011). However, the patterns of variation are difficult to unravel as they are influenced by both the genome and the environment (Labarrere et al., 2019). The causes of intraspecific variation may be a response to environmental variation (heterogeneity), leading to phenotypic plasticity. The contribution of plant-environment interactions to the secondary metabolite synthesis, has already been demonstrated in many plant species; *Pilocarpus pennatifolius* Lem (Allevato et al., 2019), *Carthamus tinctorius* L (Zeng et al., 2022), *Epilobium hirsutum* L. and *E. parviflorum* Schreb (Mohammadi Bazargani et al., 2021). Intraspecific variation can also be the result of genetic differences due to local adaptation. The selection pressure to which populations are exposed by colonizing a wide range of environmental conditions can result in genetic differentiation in important traits, including the accumulation of specialized metabolites (Kawecki and Ebert, 2004; Savolainen et al., 2007). The approach that makes it possible to identify the contributions of genetics and environment to phenotypic variation is a field experiment in which individuals from different populations are grown under uniform environmental conditions (Conover and Schultz, 1995; De Villemereuil et al., 2015).

One of the best-known insecticidal phytochemicals is pyrethrin, a specialized metabolite from Dalmatian pyrethrum (*Tanacetum cinerariifolium* (Trevis.) Sch. Bip.). Pyrethrins are extracted from dried and ground flower heads because approximately 94% of the total pyrethrins accumulate there (Head, 1966). More precisely, the pericarp of the achenes is the part of the flower where the highest concentrations are found. The achenes are densely covered with glandular trichomes, which have the main function in the biosynthesis of pyrethrins (Ramirez et al., 2012).

Pyrethrin consists of six compounds (pyrethrin I and II, cinerin I and II and jasmolin I and II) which belong to the chemical class of monoterpene esters (Casida, 1973; Crombie, 1995; Essig and Zhao, 2001). Pyrethrins I and II have different modes of action, with pyrethrin I having a lethal effect, while pyrethrin II is known to have a *knockdown* effect. Pyrethrin I is usually the most abundant component, with the average proportion in the pyrethrum extract being up to more than 50%. The ratio of the two main components, pyrethrin I and II, determines the bioactivity of the pyrethrin extract, i.e., the higher the ratio, the stronger the insecticidal effect (Maciver, 1995).

Unlike many synthetic pesticides, pyrethrin is highly biodegradable in water, sunlight and air, and despite the long history of use, resistance has very rarely been observed in practice (e.g. Yang et al., 2020). The WHO (2010) report states that the available data on humans show no significant adverse health effects following exposure to modern pyrethrin-based products. Hernández-Moreno et al. (2013) also confirmed that the pyrethrin levels used are not of concern to consumers or farmers. However, when applied directly under laboratory conditions pyrethrins are toxic to fish (Pillmore, 1973), amphibians (Oliveira et al., 2019) and aquatic invertebrates (e.g. crayfish) (Camougis and Davis, 1971). The detailed overview of pyrethrins impact on the environment, toxicity on the target and non-target species, as well as biosynthesis, biological activity, extraction and quantification methods and factors influencing pyrethrin content and composition (morphological characteristics, flower maturity/harvest time, agrotechnical practices, etc.) can be found in the article by (Jeran et al., 2020) and the references provided.

Dalmatian pyrethrum occurs naturally in the eastern Mediterranean (Eu-Mediterranean and Sub-Mediterranean zones) and is a common component of low-growing shrub vegetation on calcareous soils. Its main distribution area includes the Croatian coastal region and islands (i.e. Dalmatia), the southern parts of Bosnia and Herzegovina and the coastal regions of Montenegro and Albania (Euro+Med, 2006; Nikolić, 2020). Historically, Dalmatian pyrethrum was cultivated in large quantities in Croatia (former Yugoslavia) (Ožanić, 1955; Benić Penava, 2012). However, production was abandoned decades ago and commercial cultivation of the species for the extraction of pyrethrins was established in the highlands in tropical climates (e. g. Kenya, Uganda) and in cool temperate climates at low altitudes (Tasmania) (Fulton, 1998). The main Dalmatian pyrethrum producing countries in 2021 were Tanzania, Rwanda, and Papua New Guinea (FAOstat, 2023). As a typical Mediterranean species, natural Dalmatian pyrethrum populations are exposed to numerous environmental stresses throughout the year, including drought stress during the hot and dry summers and frost stress due to the characteristic temperature drop in winter (Morales et al., 2016). The Mediterranean basin is highly vulnerable to the negative effects of climate change and, in particular, to fluctuations in precipitation patterns and the occurrence of extremely high temperatures. These two interacting unfavorable environmental conditions will certainly have a detrimental effect on many plant species. Considering all the risks to which plant species are exposed due to climate change, knowledge of the role of secondary metabolites in their adaptability to stressful conditions is crucial. Studying the traits that are subjected to selection and the associated bioclimatic factors responsible for it is an important step towards understanding local adaptation (Kawecki and Ebert, 2004). Recent studies (Grdiša et al., 2013; Varga et al., 2021), have shown high variability in pyrethrin content and composition in natural Dalmatian pyrethrum populations. As an addition to the previous studies, we distinguished here for the first time whether these differences are the result of adaptation to the native environment in which they evolved or a short-term response to environmental conditions. The specific objectives of this study were therefore: (1) to quantify genetically determined phenotypic differentiation in 15 natural Dalmatian pyrethrum populations grown under the same conditions in a field trial, (2) to define bioclimatic factors associated with the accumulation of individual pyrethrin compound and (3) to investigate relative contribution of bioclimatic factors to the local adaptation of natural Dalmatian pyrethrum populations. The study improves the understanding of the local adaptation of the species and provides important information for its conservation and use in breeding new cultivars adapted to ongoing climate change.

## 2 Materials and methods

### 2.1 Collection of plant material and field experiment

The seed accessions of the natural populations of Dalmatian pyrethrum were collected along the species’ natural distribution range in Croatia, Bosnia and Herzegovina and Montenegro. Accessions are stored and maintained as part of the Collection of Medicinal and Aromatic Plants (available at: https://cpgrd.hapih.hr/) of the National Gene Bank of the Republic of Croatia, in the Department of Plant Biodiversity, Faculty of Agriculture of the University of Zagreb. A total of 15 populations were collected; one island population from the northern Adriatic (P01), five populations from the central Adriatic (P02-P06), three island populations from the southern Adriatic (P07-P09), and one southern Adriatic coastal population (P10 Srđ). Seeds were also sampled from a population inhabiting southern parts of Bosnia and Herzegovina (P11), and four inland populations from the western and southwestern parts of Montenegro (P12-P15). The voucher specimens are kept in the ZAGR Herbarium Collection, Faculty of Agriculture, University of Zagreb (available at: http://herbarium.agr.hr/). The populations spanned an elevation gradient from 14 to 1031 m above sea level. The exact sampling locations and associated data are shown in **Table 1**. To characterize the climatic conditions at the sampling sites (native environments), 19 temperature and precipitation-related bioclimatic variables (BIO01-BIO19) were extracted from the WorldClim database (available at: www.worldclim.org) (**Supplementary Table S1**).

**Table 1:** Detail information on the sampling sites of Dalmatian pyrethrum populations.

Pop	Location	Country	Accession Number^a^	Herbarium ID^b^	Latitude (N)^c^	Longitude (E)^c^	Altitude (m a.s.l.)
P01	Vrbnik, Krk	HRV	MAP02970	ZAGR-76570	45.07	14.66	100
P02	Telašćica, Dugi otok	HRV	MAP02961	ZAGR-76571	43.90	15.14	75
P03	Žman, Dugi otok	HRV	MAP02959	ZAGR-76574	43.94	15.14	85
P04	Ugljan	HRV	MAP02971	ZAGR-76575	44.08	15.19	38
P05	Murter	HRV	MAP02796	ZAGR-76576	43.78	15.65	22
P06	Sevid	HRV	MAP02965	ZAGR-76577	43.50	16.04	159
P07	Vis	HRV	MAP02973	ZAGR-76578	43.07	16.25	16
P08	Korčula	HRV	MAP02824	ZAGR-76579	42.95	17.12	118
P09	Mljet	HRV	MAP02964	ZAGR-76585	42.78	17.33	14
P10	Srđ	HRV	MAP02966	ZAGR-76586	42.65	18.11	382
P11	Trebinje	BIH	MAP02969	ZAGR-76587	42.72	18.30	507
P12	Grahovo	MNE	MAP02977	ZAGR-76588	42.67	18.65	856
P13	Lovćen	MNE	MAP02979	ZAGR-76589	42.37	18.88	1031
P14	Budva	MNE	MAP02976	ZAGR-76590	42.32	18.89	772
P15	Vranjske Njive, Podgorica	MNE	MAP02975	ZAGR-76591	42.47	19.25	61

a Accession number from the Croatian Plant Genetic Resources Database.

b Herbarium ID number assigned to voucher specimens in the ZAGR Herbarium.

c Latitude and longitude are expressed in WGS84S96 coordinate reference system.

To exclude the influence of environmental factors on the variability of pyrethrin content and composition, field trials were conducted under uniform conditions at the experimental station Maksimir of the Department of Plant Biodiversity of the Faculty of Agriculture of the University of Zagreb (45.83° N, 16.03° E). The individual plants were grown from seed in a greenhouse and transplanted to the experimental station after about 90 days of growth. A row-column experimental design with two replicates was used, with each population (15) represented by 20 individual plants (300 plants in total). The distance between the rows was 120 cm and the plant spacing in a row was 100 cm.

### 2.2 Ultrasound-assisted extraction of pyrethrin

The flower heads of each individual plant were collected 14 months after transplanting to the experimental station, in FS4 (2–5 rows of open disc flowers) as suggested by Grdiša et al. (2022). For the extraction of pyrethrins, the ultrasound-assisted extraction described in Babić et al. (2012) was used. The extraction was performed with 0.25 g of powdered flower heads in an ultrasonic bath (Labsonic LBS2–10, FALC, Treviglio, Italy) using acetone (5 ml) as extraction solvent. The temperature applied was 50°C for a period of 60 minutes. The extracts were filtered through a 0.45 μm pore diameter filter (HPLC certified, Pall Life Sciences, Port Washington, NY). Extracts for each sample (300) were carried out in duplicate.

### 2.3 High performance liquid chromatography

The qualitative and quantitative determination of the individual pyrethrin compound was carried out as in Grdiša et al. (2022) using high-performance liquid chromatography coupled with DAD-UV/VIS (Agilent Technologies, Santa Clara, CA, USA). The separation was performed on Zorbax SB C18 250 x 4.6 mm particle size 5 μm (Agilent Technologies, Santa Clara, CA, USA) in gradient elution mode, where the mobile phase used in the chromatographic system was 0.1% phosphoric acid in MilliQ water (A) and acetonitrile (B) (**Table 2**) The flow rate of the mobile phase was 1.4 ml/min, the injection volume was 5 μl and the detection was carried out at 225 nm. Injections were performed in duplicate for each sample. Chromatogram of Dalmatian pyrethrum extract is shown in **Supplementary Figure S1**.

**Table 2:** HPLC- DAD-UV/VIS mobile phase gradient elution conditions.

Time (min)	A (%)	B (%)
0.00	40	60
15.00	40	60
25.00	20	80
35.00	20	80
35.10	40	60
40.00	40	60

### 2.4 Data analysis

Analysis of variance (ANOVA) was used to compare six pyrethrin compounds, total pyrethrin content, and pyrethrin I/pyrethrin II ratio by population, followed by Tukey’s HDS tests. For this purpose, the GLM procedure in SAS v9.4 (SAS Institute, 2004) was used. Arcsine transformations were performed for all percentage data. Pearson correlation coefficients were calculated between six pyrethrin components, total pyrethrin content and the pyrethrin I/pyrethrin II ratio, as well as between 19 bioclimatic variables using the CORR procedure in SAS. The PRINCOMP procedure in SAS v9.4 was used to perform a principal component analysis (PCA) for 19 bioclimatic variables. The Kaiser criterion and scree plot was used to determine the number of principal components that should be retained to produce a biplot showing the populations and bioclimatic variables (as vectors). The population scores of the first three principal components were used in the cluster analysis (CA). The CLUSTER procedure in SAS v9.4 was applied to perform the UPGMA clustering of the populations and determine the optimal number of bioclimatic groups based on the Cubic Clustering Criterion (CCC) and the Pseudo-F (PSF) statistic. The principal component analysis for six pyrethrin components was performed in the same way as for the bioclimatic data.

## 3 Results

### 3.1 Content and composition of pyrethrin in Dalmatian pyrethrin populations

The variance analysis (ANOVA) showed that the content and composition of pyrethrins varied significantly among populations (**Table 3** **).** The significantly highest average content of pyr I (66.47% of total pyrethrin - TP) and the significantly lowest average pyr II content (22.91% TP) were detected in the population P15 Vranjske Njive. In contrast, the population P04 Ugljan was the population with the lowest average pyr I content (34.04% TP) and the highest pyr II content (51.56% TP). The lowest average values for cin I were determined in P05 Murter (2.67% TP) and the highest in P10 Srđ (5.56% TP). The highest observed value of the cin II content (7.27% TP) was found in P4 Ugljan and this population also had the lowest levels of jas I (1.23% TP). The lowest content of cin II was found in P15 Vranjske Njive (1.41% TP), while this population had the highest amount of jas I (4.37% TP). Population P06 Sevid had the highest amount of jas II (2.14%) and P10 Srđ the lowest amount of this component (1.32%). In eight out of 15 populations, total pyrethrin content (expressed as % of flower dry weight - DW), was greater than 1%. The highest total pyrethrin content was found in P14 Budva (1.27%), followed by P08 Korčula (1.15%), P15 Vranjske Njive (1.14%) and P01 Vrbnik, Krk (1.13%). The significantly highest pyr I/pyr II ratio (3.10) was found in P15 Vranjske Njive.

**Table 3:** The content of six pyrethrin compounds (expressed as % of total pyrethrin - TP), the total pyrethrin content (% of flower dry weight - DW) and the pyrethrin I/pyrethrin II ratio in 15 Dalmatian pyrethrum populations.

ID	Pyrethrin I^*^ (%)	Pyrethrin II (%)	Cinerin I (%)	Cinerin II (%)	Jasmolin I (%)	Jasmolin II (%)	Total pyrethrin content (%)	Pyrethrin I/Pyrethrin II Ratio
P01	52.91 ± 10.44^b^	36.11 ± 9.86^cde^	3.07 ± 0.95^bc^	2.85 ± 1.45^f^	2.94 ± 1.00^b^	2.12 ± 0.70^a^	1.13 ± 0.24^abc^	1.70 ± 0.99^bc^
P02	39.34 ± 6.85^de^	47.62 ± 6.84^ab^	4.05 ± 1.23^abc^	6.03 ± 1.87^ab^	1.29 ± 0.43^fg^	1.68 ± 0.29^ab^	0.95 ± 0.18^bcd^	0.87 ± 0.33^cd^
P03	50.76 ± 9.77^bc^	37.71 ± 10.10^bcde^	3.93 ± 1.47^abc^	3.54 ± 1.58^def^	2.29 ± 0.78^bcde^	1.77 ± 0.44^ab^	0.92 ± 0.22^bcd^	1.55 ± 0.83^bcd^
P04	34.04 ± 7.82^e^	51.56 ± 8.83^a^	4.13 ± 1.75^abc^	7.27 ± 1.99^a^	1.23 ± 0.41^g^	1.78 ± 0.33^ab^	0.87 ± 0.22^bcd^	0.70 ± 0.25^d^
P05	45.12 ± 11.09^bcd^	45.49 ± 10.92^abc^	2.67 ± 1.23^c^	3.35 ± 1.69^ef^	1.68 ± 0.56^efg^	1.69 ± 0.59^ab^	1.01 ± 0.38^abcd^	1.16 ± 0.76^bcd^
P06	42.20 ± 10.86^cde^	44.38 ± 10.90^abcd^	4.13 ± 1.59^abc^	5.33 ± 1.85^abc^	1.84 ± 0.55^def^	2.14 ± 0.66^a^	0.86 ± 0.28^d^	1.08 ± 0.59^bcd^
P07	52.10 ± 10.81^b^	35.43 ± 9.33^cde^	3.98 ± 1.43^abc^	3.50 ± 1.70^ef^	2.93 ± 0.73^b^	2.07 ± 0.78^a^	1.09 ± 0.40^abcd^	1.66 ± 0.79^bc^
P08	48.96 ± 9.05^bcd^	36.47 ± 10.57^cde^	5.32 ± 2.75^a^	4.37 ± 1.46^bcdef^	2.75 ± 0.76^bc^	2.12 ± 0.68^a^	1.15 ± 0.34^abc^	1.63 ± 1.18^bc^
P09	50.84 ± 6.54^bc^	36.75 ± 5.62^cde^	4.58 ± 1.38^ab^	4.26 ± 1.69^bcdef^	2.04 ± 0.47^cde^	1.53 ± 0.52^ab^	0.82 ± 0.15^d^	1.44 ± 0.41^bcd^
P10	49.76 ± 11.60^bc^	36.36 ± 10.58^cde^	5.56 ± 1.61^a^	5.30 ± 2.36^bcd^	1.71 ± 0.56^efg^	1.32 ± 0.49^b^	1.08 ± 0.17^abcd^	1.74 ± 1.48^bc^
P11	52.70 ± 10.70^b^	34.88 ± 10.31^de^	4.48 ± 1.43^ab^	3.70 ± 1.66^cdef^	2.48 ± 0.89^bcd^	1.76 ± 0.72^ab^	1.05 ± 0.17^abcd^	1.78 ± 1.04^b^
P12	53.36 ± 6.72^b^	33.57 ± 8.33^e^	5.12 ± 1.55^a^	4.29 ± 1.28^bcdef^	2.26 ± 1.02^bcde^	1.39 ± 0.34^b^	0.95 ± 0.19^bcd^	1.78 ± 0.87^b^
P13	47.78 ± 7.88^bcd^	39.43 ± 7.92^bcde^	4.32 ± 1.44^ab^	4.45 ± 1.47^bcde^	2.22 ± 0.75^bcde^	1.81 ± 0.66^ab^	0.87 ± 0.26^cd^	1.32 ± 0.60^bcd^
P14	48.34 ± 6.63^bcd^	37.87 ± 7.32^bcde^	4.79 ± 1.30^a^	4.49 ± 1.18^bcde^	2.42 ± 0.60^bcd^	2.10 ± 0.50^a^	1.27 ± 0.16^a^	1.36 ± 0.46^bcd^
P15	66.47 ± 5.01^a^	22.91 ± 5.35^f^	3.22 ± 1.50^bc^	1.41 ± 0.71^g^	4.37 ± 0.89^a^	1.62 ± 0.35^ab^	1.14 ± 0.22^ab^	3.10 ± 0.94^a^

*Average ± standard deviation based on 20 individual plants. Values denoted by the same letter are not significantly different at P < 0.05.

As an indicator of variability, the coefficient of variation (CV %) was determined both at the compound level and at the population level. Among the pyrethrin compounds, the variability was greater for the cinerins and jasmolins, with the highest CV values determined for cin II and jas I, 48.79% and 44.94%, respectively. The greatest consistency was found for the pyr I content (CV = 23.07%) (**Supplementary Table S2** **).** At the population level, the highest degree of variability in the content of pyr I was detected in P06 Sevid (25.72%), where the content between individuals ranged from 21.00 to 63.41% TP, and the lowest in P15 Vranjske Njive, Podgorica (CV = 7.54%, range: 56.41–73.52%). For pyr II content, the highest variability among individuals was observed in population P11 Trebinje (29.56%) and the lowest in P02 Telašćica, Dugi otok (14.37%). The range of total pyrethrin content was the widest in P05 Murter (0.22–1.85), resulting in a coefficient of variation of 37.25%. For total pyrethrin content, the lowest average variability was observed in P014 Budva (12.28%). The population with an extremely high coefficient of variation for the observed pyr I/pyr II ratio was population P10 Srđ (85.2%) (**Supplementary Table S3** **).**

### 3.2 Correlations of the six pyrethrin compounds

**Figure 1:**
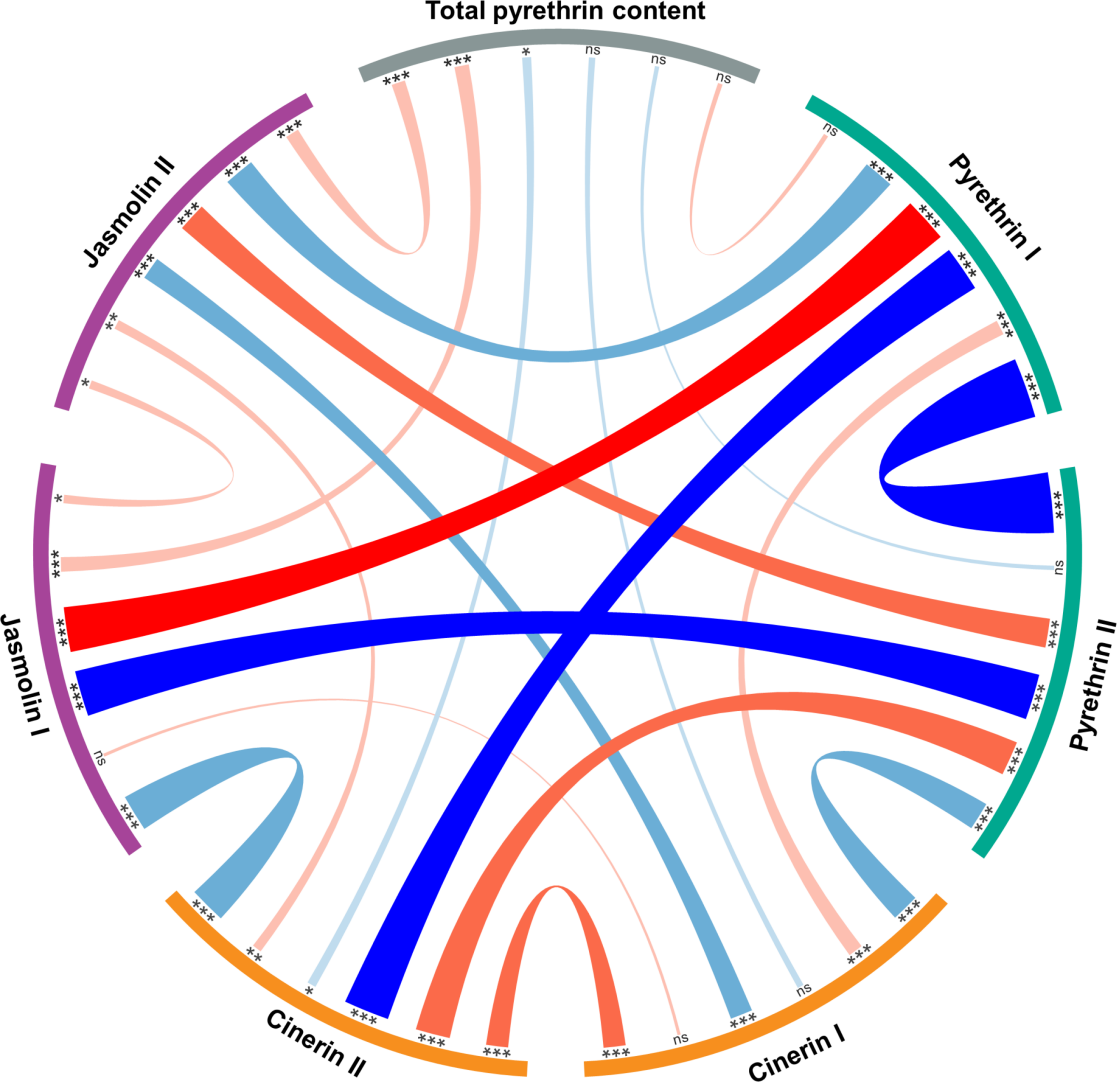
Circos diagram showing Pearson correlation coefficients between six pyrethrin compounds (expressed as % of total pyrethrin), total pyrethrin content (% of flower dry weight) and pyrethrin I/pyrethrin II ratio. *ns - non-significant; * - significant at *P* < 0.05; ** - significant at *P* < 0.01; *** - significant at *P* < 0.001.

The Pearson correlation was calculated to measure the relationship between the six pyrethrin compounds and the total pyrethrin content as well as the pyr I/pyr II ratio. A strong positive (*r* > 0.70) and significant correlation was found between pyr I and jas I (*r* = 0.718, *P* < 0.001). The strong (< -0.70) significant negative correlations were observed between pyr I and pyr II (*r* = -0.966, *P* < 0.001), pyr II and jas I (*r* = -0.734, *P* < 0.001), as well as pyr I and cin II (*r* = -0.731, *P* < 0.001). The results are shown in a circos diagram (**Figure 1** **).**

### 3.3 Identification of bioclimatic groups

The environmental data from the sampling sites (**Supplementary Table S1**) consisted of 19 temperature and precipitation variables some of which were strongly correlated (**Supplementary Table S4**) and were subjected to dimensionality reduction by principal component analysis (PCA). The three PCs were extracted that explained 93.13% of the total variation in bioclimatic conditions at the sampling sites. Correlation analysis revealed that PC1 (explaining 65.21% of the total variance) was significantly and highly negatively correlated with temperature variables (BIO01, BIO05, BIO06 and BIO08-BIO11) (*r* = < -0.70, *P* < 0.001) and positively correlated with precipitation variables (BIO12-BIO19) (*r* = > 0.70, *P* < 0.001). PC2, which explained 20.91% of the total variance, was significantly and strongly positively correlated with the temperature variables (BIO02, BIO04, BIO07) (*r* = > 0.70, *P* < 0.001) (**Supplementary Table S5** **).**

**Figure 2:**
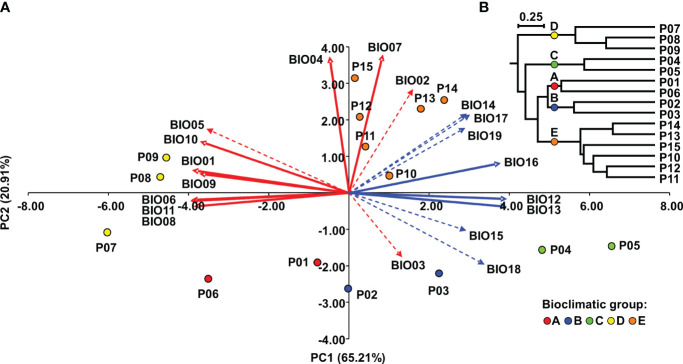
Bioclimatic groups of Dalmatian pyrethrum populations: **(A)** Biplot of principal component analysis of Dalmatian pyrethrum populations based on 19 bioclimatic variables. Red vectors represent temperature-related variables and blue vectors precipitation-related variables. The dashed vectors represent variables for which the correlation with PC1 and PC2 is between *r* = -0.70 and 0.70. **(B)** UPGMA dendrogram of Dalmatian pyrethrum populations based on population scores of the first three principal components.

**Figure 3:**
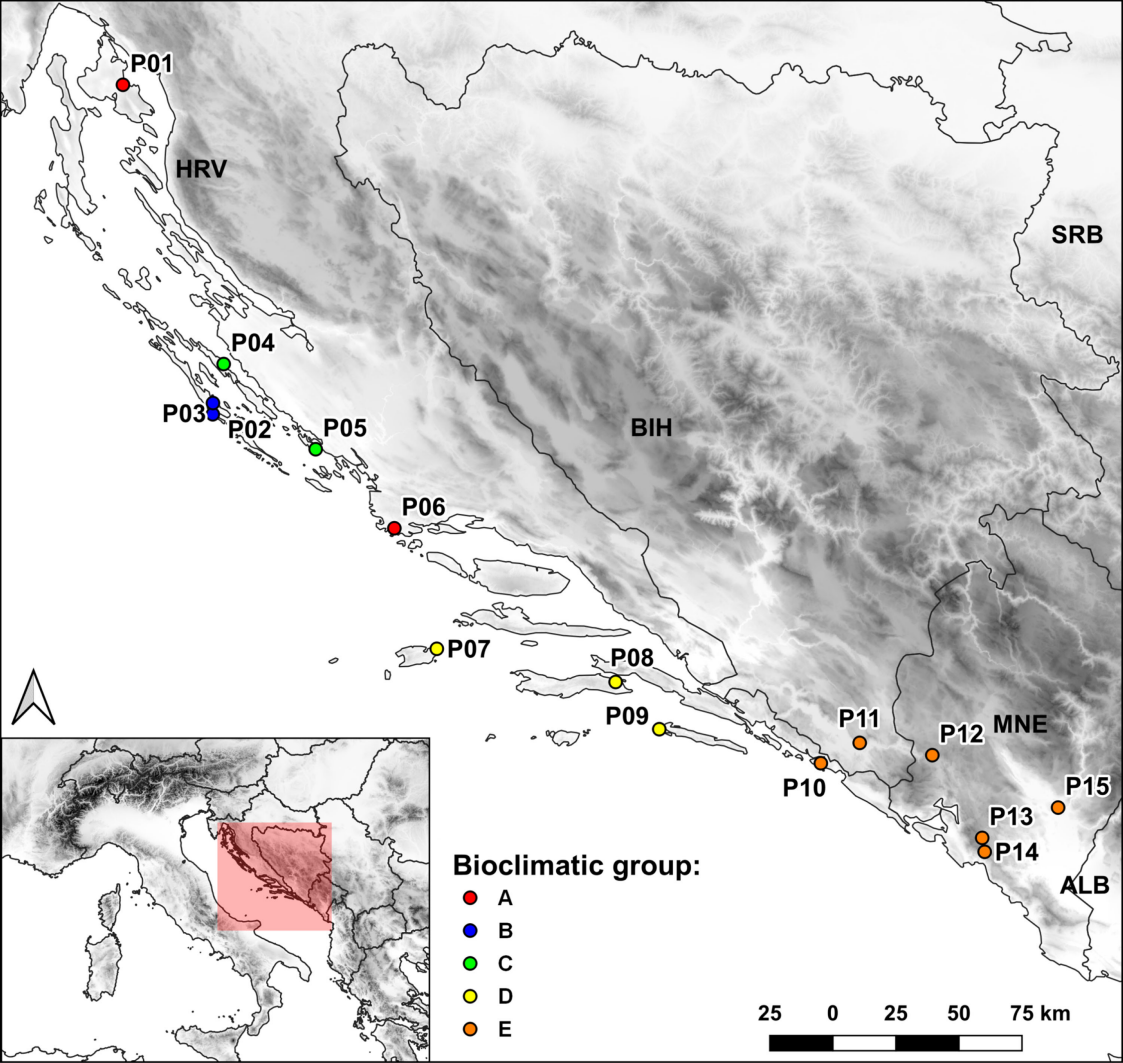
Map of the sampled populations and bioclimatic groups to which they were assigned based on cluster analysis.

The PC plot (**Figure 2A**) shows the grouping of the Dalmatian pyrethrum populations according to the environmental similarities of their sampling sites. To classify populations into bioclimatic groups based on 19 temperature and precipitation variables, a UPGMA clustering algorithm was applied to population scores of the first three principal components. The populations were classified into five bioclimatic groups, referred to as A, B, C, D, and E (**Figure 2B** **),** based on Cubic Clustering Criterion (CCC) and the Pseudo-F (PSF) statistics. On PC plot the populations are colored according to the bioclimatic group to which they were assigned in the cluster analysis. The most pronounced separation along PC1 was that of the populations of the southern Adriatic islands (P07 Vis, P08 Korčula, P09 Mljet; subsequently assigned to bioclimatic group D) whose sampling sites were characterized by higher values for temperature-related variables that indicate higher temperatures; and lower precipitation levels, and the populations sampled from the southernmost part of the sampling area (P10 Srđ, P11 Trebinje, P12 Grahovo, P13 Lovćen, P14 Budva and P15 Vranjske Njive, Podgorica; subsequently assigned to bioclimatic group E), which were characterized by largest temperature fluctuations as revealed from wider temperature annual range (BIO07), mean diurnal range (BIO02), and temperature seasonality (BIO04) variables. A further separation of P04 Ugljan and P05 Murter (subsequently assigned to as bioclimatic group C) along PC1 was based on the greatest annual precipitation (BIO12) and the precipitation of the wettest month (BIO13) and wettest quarter (BIO16). Along PC2 the populations from Dugi otok (P02 and P03; subsequently assigned to bioclimatic group B) were separated from the southernmost populations based on the variable BIO03 indicating considerable isothermality on the island of Dugi otok. Populations P01 Vrbnik, Krk and the inland population P06 Sevid from the central Adriatic (subsequently assigned to bioclimatic group A) were the most similar in terms of environmental conditions with the populations assigned to bioclimatic group B. These two groups were placed opposite to vectors for temperature seasonality (BIO04) and temperature annual range (BIO07), implying that they experience substantially lower seasonal variation in temperature compared with the populations belonging to group E (P10-P15). **Figure 3** shows the populations studied with their bioclimatic group assignment in different colors.

### 3.4 Pyrethrin diversity

**Figure 4:**
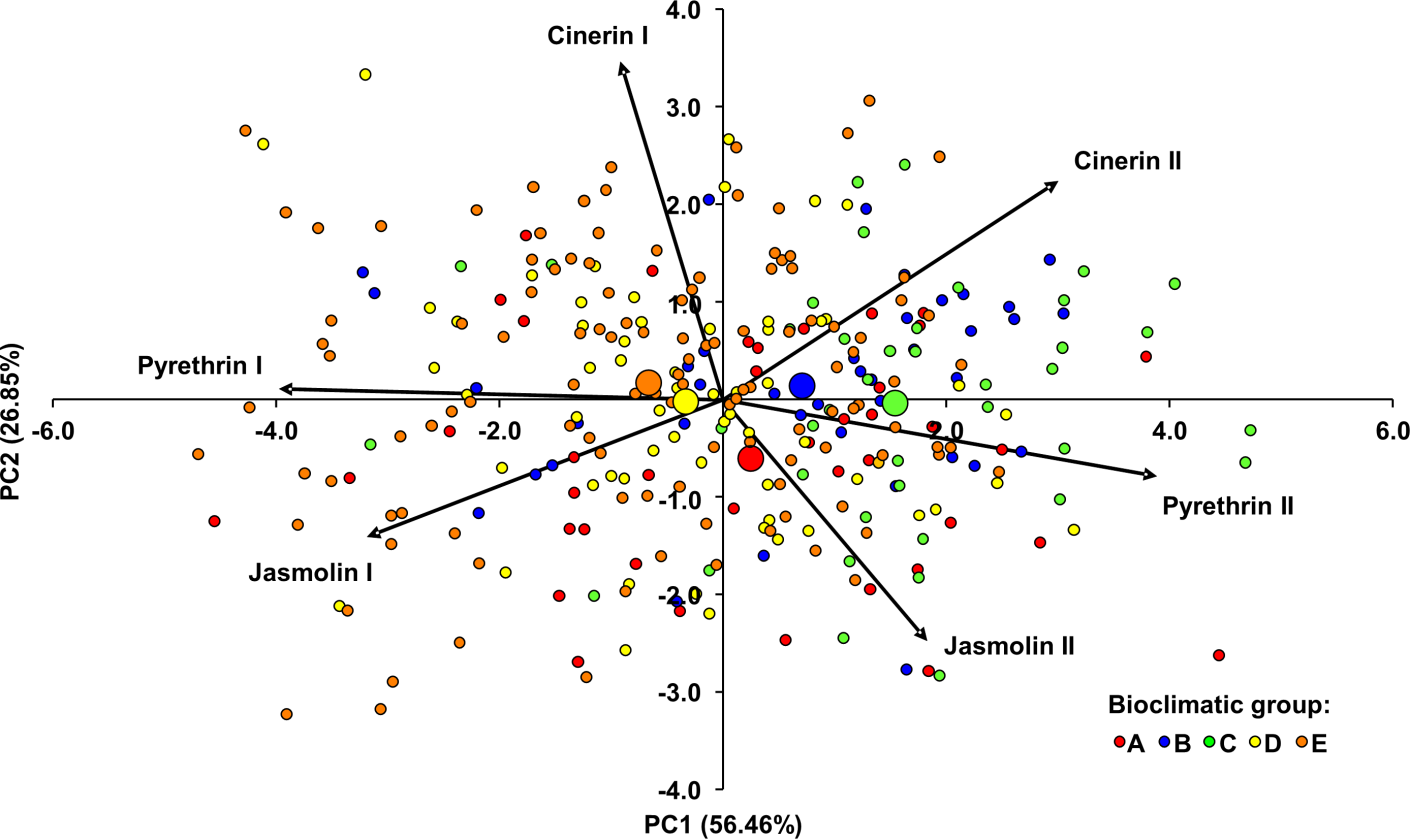
Principal component analysis biplot of the Dalmatian pyrethrum samples based on six pyrethrin compounds. The individual plants were assigned to five bioclimatic groups (A–E). The barycenters of the bioclimatic groups are represented by larger circles.

Principal component analysis (PCA) was also performed to examine and visualize similarities in the composition of the pyrethrin extract (**Figure 4** **).** It was performed for all individuals from 15 populations by using the contents of the six pyrethrin compounds as variables. The dimensionality of the data was reduced from six partially correlated variables to two uncorrelated principal components, accounting for 83.31% of the observed variation. The eigenvalues and percentages of cumulative variance are shown in **Supplementary Table S6**. PC1 was mainly determined by pyr I and pyr II. It was negatively correlated with pyr I (*r* = -0.993, *P* < 0.001) and positively correlated with pyr II (*r* = 0.971, *P* < 0.001). A strong significant correlation was also found between PC1 and cin II (*r* = 0.749, *P* < 0.001) and with jas I (*r* = -0.797, *P* < 0.001). The most influential parameter for PC2 was cin I, which showed a strong significant correlation with this PC (*r* = 0.867, *P* < 0.001). **Figure 4** shows the projection of the individuals and the barycenters of the bioclimatic groups onto the plane defined by the two PCs. Most of individuals were scattered in relation to the barycenters of the bioclimatic groups to which they were assigned, suggesting high variability within the bioclimatic groups. In general, PC1 separated the individuals/bioclimatic groups according to the content of pyr II and pyr I, separating bioclimatic groups B and C (characterized by the highest content of pyr II) from bioclimatic groups D and E in which a higher content of pyr I was found. Along PC2, bioclimatic group A, as the one with the highest content of jas II was separated from the other groups.

### 3.5 Variability of pyrethrin content and composition in bioclimatic groups

**Figure 5:**
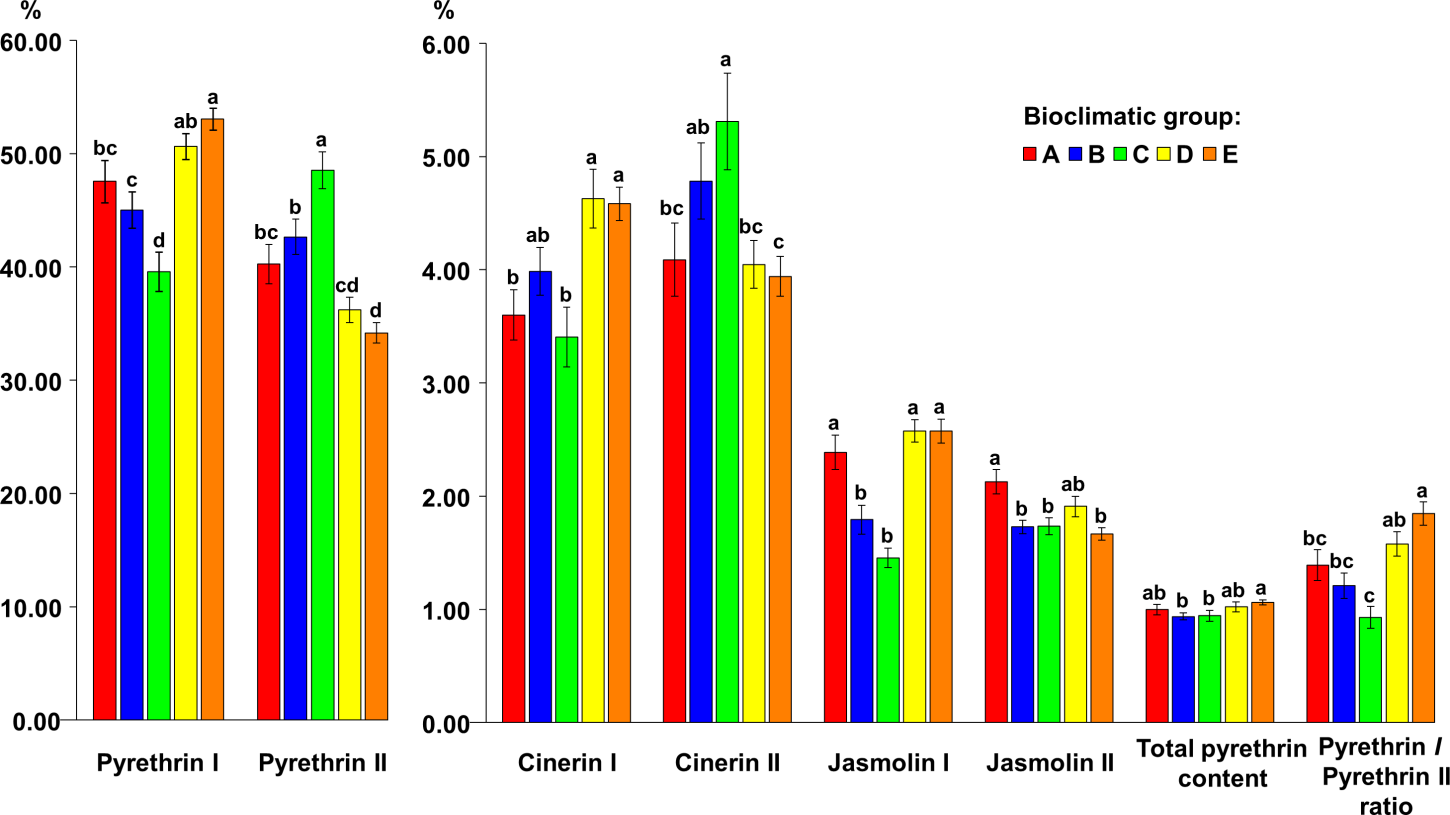
The content of six pyrethrin compounds (expressed as % of total pyrethrin), the total pyrethrin content (% of flower dry weight) and the pyrethrin I/pyrethrin II ratio in five bioclimatic groups of Dalmatian pyrethrum populations. Different letters indicate significant differences between the bioclimatic groups at *P* < 0.05.

Bioclimatic group E was characterized by the highest average values for pyrethrin I (53.07% TP), total pyrethrin content (1.06% flower DW) and pyr I/pyr II (1.85) but did not differ significantly in these parameters from bioclimatic group D (pyr I = 50.63% TP; TP = 1.02% flower DW; pyr I/pyr II = 1.58), and in total pyrethrin content from bioclimatic group A (1.00% flower DW). However, significant differences were found compared to the other bioclimatic groups. Bioclimatic group C had the significantly highest portion of the pyr II content (48.52% TP) and cin II (5.31% TP), followed by bioclimatic group B (pyr II = 42.66% TP, cin II = 4.78% TP). A significant difference in cin I content was found between bioclimatic groups A/C vs. D/E, while bioclimatic group D had the highest average content of this compound (4.63% TP). The highest average content of cin II was found in bioclimatic group C (5.31% TP), followed by bioclimatic group B (4.78% TP). The difference in cin II content between these two groups was not significant. The jas I content did not differ significantly between A, D and E, however, it was significantly lower in bioclimatic groups B and C. The jas II content was highest in bioclimatic group A (2.13% TP) and it was significantly higher compared to bioclimatic groups B, C and E (**Figure 5** **).**

To compare the variability in pyrethrin content between the significantly different bioclimatic groups, the coefficient of variation was calculated. Greater constancy was found for pyr I and II in comparison to the other four pyrethrin compounds. Among the bioclimatic groups, the highest variability of the pyr I content (CV = 27.81%), as well as pyr I/pyr II ratio (CV = 65.00%), was found in bioclimatic group C and the lowest for group D (CV = 17.61% and 53.35%), while the pyr II content was the most variable in bioclimatic group E (CV = 29.01%). A high rate of variability was observed for cin II in bioclimatic group A as shown by the calculated coefficient of variation (CV = 50.76%). The total pyrethrin contents were heterogeneous between the bioclimatic groups, ranging from CV = 21.59% (bioclimatic group B, in which the total pyrethrin content was between 0.33 and 1.37% flower DW) to CV = 33.16% (bioclimatic group D). Within this bioclimatic group, total pyrethrin levels were found to range from 0.60–1.94% flower DW (**Supplementary Table S3**).

### 3.6 Associations between environmental variations and pyrethrin diversity

A weak but moderately to highly significant correlation was found between some of the bioclimatic variables and the content of pyrethrin compounds. The levels of pyr I and jas I were positively correlated with BIO04 Temperature Seasonality (*r* = 0.412, *P* < 0.001 and *r* = 0.425 P < 0.001.), and negatively correlated with BIO03 Isothermality (*r* = -0.355 P < 0.001 and *r* = -0.426 P < 0.001). The pyr I/pyr II ratio followed the same pattern as pyr I and jas I, showing a significantly positive correlation with BIO04 Temperature Seasonality (*r* = 0.348, *P* < 0.001) and a negative correlation with BIO03 isothermality (*r* = -0.290, *P* < 0.001). The situation was reversed for pyr II, where a negative correlation with BIO04 Temperature Seasonality (*r* = -0.428, *P* < 0.001) and s positive correlation with BIO03 Isothermality (*r* = 0.353, *P* < 0.001) was found. A weak but positive correlation (significant) was also found between pyr II and BIO15 Precipitation seasonality (*r* = 0.207, *P* < 0.001) and BIO18 precipitation of the warmest quarter (*r* = 0.345, *P* < 0.001). (**Supplementary Table S7**).

## 4 Discussion

In this study, we conducted a geographically comprehensive sampling of the Dalmatian pyrethrum populations throughout the entire range within the Mediterranean region, where this plant species occurs naturally. The Mediterranean region is known as a mosaic of biodiversity-rich ecosystems, characterized by a wide environmental heterogeneity and numerous vegetation types (Médail, 2016) which are interconnected in a complex pattern shaped by multiple abiotic and biotic factors. Studies that examine specialized metabolites in plants suggest that these biotic and abiotic influences can have a considerable impact on the qualitative and quantitative diversity of a species (Allevato et al., 2019), which might reflect genetic adaptation or short-term response to environmental conditions (Spitaler et al., 2006). To identify patterns consistent with local adaptation, we tested whether each of the 19 bioclimatic variables (recorded at the original growing site) influenced the pyrethrin content and composition measured in the field trial.

### 4.1 Variability in pyrethrin content and composition within and among Dalmatian pyrethrum populations

The results obtained show considerable biochemical variability among and within Dalmatian pyrethrum populations. However, some populations show lower variability for some pyrethrin compounds, e. g. pyrethrin I in populations from Montenegro (P12-P15). In most of the populations analyzed (10) pyrethrin I was the dominant component. Although all six components interact synergistically to achieve an insecticidal effect, pyrethrin I and II are typically present in the highest amount in the pyrethrum extract, mainly pyrethrin I, which is a very lethal and fast-acting compound. In the study by Tattersfield et al. (1929), pyrethrin I was found to be about ten times more toxic to the insects tested (e.g., *Aphis rumicis* Linnaeus, 1758) than pyrethrin II, which was found to have a *knockdown* effect (Sawicki et al., 1962). Therefore, the extract with a high content of pyrethrin I and a higher pyrethrin I/pyrethrin II ratio is preferred. To demonstrate the insecticidal potential of the pyrethrum extracts, the ratio between these two components was also calculated and a range of 0.87 (P02 Telašćica, Dugi otok) to 3.10 (P15 Vranjske Njive, Podgorica) was found, which is as high as in some breeding lines (0.47 to 3.5) (Bhat, 1995). The total pyrethrin content ranged from 0.82% flower DW (P09 Mljet) to 1.27% flower DW (P14 Budva). Compared to high-yielding commercial cultivars, developed through long artificial selection processes in Australia, Kenya, and the USA, which were found to have much higher total pyrethrin content (more than 3%), the total pyrethrin content in this study was rather low, but to be expected in natural populations. Previous studies on natural populations found similar values for the total pyrethrin content: 0.36 to 1.30% (Grdiša et al., 2022), 1.1–1.3% (Ban et al., 2010), and 1–1.2% (Babić et al., 2012). In the more recent study by (Varga et al., 2021), a lower average pyrethrin content of 0.22 to 0.87% was reported, which could be due to the different extraction method used.

Since the variability in pyrethrin content and composition persisted among and within populations of Dalmatian pyrethrum under uniform growing conditions (field trial experiment), we can conclude that the variability is the result of underlying genetic divergence. Several studies have shown that the synthesis and accumulation of secondary metabolites contributes to local adaptation. For example, in their common garden study on *Artemisia californica* Pratt et al. (2014) showed genetically determined variation in terpene composition and monoterpene concentration correlated with the latitude of the original growing location of the source population and corresponding differences in rainfall. In their study Zhou et al. (2021) found a positive correlation between the organic acid content in *Agriophyllum squarrosum* and the environmental variables (altitude, latitude, longitude, temperature, and precipitation) of the original growing areas, suggesting they are involved in adaptation to high altitude.

### 4.2 Influence of native environment on pyrethrin content and composition

To fully characterize the range of conditions found in the natural distribution area of *T. cinerariifolium*, we used the data from the WorldClim database. The selected bioclimatic variables represent temperature and precipitation conditions which are important factors for the synthesis of specialized metabolites. We found that the climatic differences between Dalmatian pyrethrum sampling sites are mainly related to temperature seasonality and isothermality as well as precipitation amount and seasonality.

The bioclimatic groups showed differences in the content of pyrethrin compounds. The highest levels of pyrethrin I, total pyrethrin and the ration of pyrethrin I and pyrethrin II ratio, were found within bioclimatic group E, characterized by the higher values of temperature seasonality (BIO04), temperature annual range (BIO07) and mean diurnal range (BIO2). On the other hand, bioclimatic group C, which was found to have the significantly highest level of pyrethrin II and cinerin II and lowest jasmolin I, is distinguished by the variables precipitation seasonality (BIO15), precipitation of warmest quarter (BIO18) as well as annual precipitation (BIO12) and warmest quarter precipitation (BIO13). Among the 19 bioclimatic variables tested, two opposite variables, isothermality (BIO03) and temperature seasonality (BIO04), were found to be the main environmental factors affecting the level of some of the pyrethrin compounds. Consequently, the results showed a weak but significant positive correlation of the pyrethrin I and jasmolin I content and the pyrethrin I and pyrethrin II ratio with temperature seasonality (BIO04), the bioclimatic variable that has been known to have a strong influence on the expression of specialized metabolites (Molina-Montenegro and Naya, 2012). On the contrary, the content of pyrethrin I and jasmolin I and the pyrethrin I/pyrethrin II ratio were negatively correlated with isothermality (BIO03), that is, temperature homogeneity or uniformity.

According to our results, higher temperatures could also contribute to increase the concentration of pyrethrin I and jasmolin I, as significant positive correlations (although weak) were also found with most of the temperature-related variables and pyrethrin I and jasmolin I. Higher temperatures in the Mediterranean region are usually associated with water deficit representing strong abiotic factors that could potentially promote the synthesis of specialized metabolites for the plants to be able to cope with these adverse effects. To characterize critical drought stress periods, the variable precipitation of the warmest quarter (BIO18), which defines interactions between rainfall amount and temperature and estimates precipitation during the warmest three months of a year, may be useful (Berio Fortini et al., 2022). In our study, this variable was significantly negatively correlated with pyrethrin I, implying that the decrease in precipitation during the warmest period (drought stress) might stimulate its accumulation. In crop production, drought stress is considered a negative factor leading to severe yield losses. However, numerous studies on aromatic and medicinal plants from the Mediterranean region, where semi-arid conditions prevail, have shown that they synthesize higher amounts of various secondary metabolites compared to plants exposed to temperate climatic conditions (reviewed in Kleinwächter and Selmar, 2015). Additionally, the available literature provides ample evidence that a common feature of different classes of specialized metabolites is the increase in their concentrations under water-deficit conditions. There are few reports showing an increase in the total content of monoterpenes, the chemical class to which pyrethrins belong, under drought stress. For example Nowak et al. (2010), found a drought-induced increase in the total amount of monoterpenes in sage (*Salvia officinalis*), while Turtola et al. (2003) found an increase in the content of monoterpenes in *Picea abies* and *Pinus sylvestris*.

Based on the results of this study, we hypothesize that the synthesis of pyrethrin I, as the most active/important compound, could be associated with its response to environmental stress, such as thermal stress caused by a wide range of temperature fluctuations throughout the year, as well as higher temperatures and drought stress during the summer months (as inferred from the variable BIO18 precipitation of the warmest quarter). This could be achieved by a heat and drought-induced increase in pyrethrin I synthesis or by favoring the selection of genotypes with high in pyrethrin I content under such environmental conditions. Furthermore, we could speculate that the increase in pyrethrin II accumulation is related to the overall lower temperatures, isothermal conditions, and exposure to the highest and variable precipitation amounts (as indicated by the variable BIO15 precipitation seasonality). Precipitation seasonality represents the irregular distribution of precipitation during a year, as well as its amount in each episode, and is a common phenomenon in the Mediterranean region (Deitch et al., 2017). A pronounced alternation between dry and rainy periods has been shown to be a stressful factor for plants, affecting the synthesis of specialized metabolites (Qaderi et al., 2023). Therefore, we hypothesize that this may have promoted the synthesis of pyrethrin II. In the investigation by Varga et al. (2021) the synthesis of pyrethrin II was also associated with lower temperatures and greater precipitation. This was the characteristic of the sampling site of the mountain Biokovo population (1350 m a.s.l) which thrives under extreme environmental conditions for a thermophilic species such as Dalmatian pyrethrum and was characterized by a unique pyrethrin profile, with pyrethrin II being the dominant component (on average 43.18% of TP).

Considering the results presented here, the high intraspecific variability seems to be a response to the different environmental conditions of the native environment (original growing site of the source population), which might induce different adaptive mechanisms in Dalmatian pyrethrum. The high intraspecific biochemical variability found in this study suggests that natural Dalmatian pyrethrum populations are valuable resources useful for future breeding programs and at the same time implies the need for individual-based selection of plant material to be potentially used. Natural populations have great potential, as they provide a broader genetic base for breeding commercial cultivars with the aim of reducing susceptibility to various biotic and abiotic stresses (Grdiša et al., 2013, Grdiša et al., 2014; Varga et al., 2021).

According to the climate projections, the Mediterranean region will be strongly affected by climate change in the near future, manly through an increase in temperature seasonality (Yettella and England, 2018), an increase in summer temperatures (Lionello and Scarascia, 2020) and more frequent heat waves accompanied by severe droughts (Giorgi and Lionello, 2008; Spinoni et al., 2018; Grillakis, 2019). In this sense, our findings on the adaptability of local populations emphasize their breeding potential in creating cultivars resilient to climate change. This would open the possibilities for successful reintroduction of Dalmatian pyrethrum into agricultural production in the Mediterranean region, as higher temperatures and drought might have a positive impact on the synthesis of pyrethrin I.

Apart from the analyzed temperature and precipitation variables, other abiotic and biotic factors also influence the production of pyrethrin compounds. We believe that further analysis is needed to clarify their contribution to the overall variation in the chemical profiles of Dalmatian pyrethrum. Future studies should also investigate the effects of stress conditions on flower dry matter content and flower yield, as they are correlated with total pyrethrin content (Parlevliet, 1974; Bhat and Menary, 1986) Understanding the key factors that influence flower dry matter content and flower yield under commercial production conditions is necessary to develop cropping strategies that deliver consistent, high pyrethrum yields under global warming conditions.

## 5 Conclusion

We found a qualitative variability of pyrethrin compounds within and between natural Dalmatian pyrethrum populations grown under uniform environmental conditions. Part of the observed variance could be explained by the environmental dataset, suggesting a relationship between some bioclimatic variables and biochemical profiles, and therefore could be considered as a result of local adaptation. The assessment of divergence between populations could support the development of conservation programs for Dalmatian pyrethrum. In addition, the results could facilitate the selection of individuals or populations for breeding programs aimed at breeding cultivars with desirable biochemical traits and adaptations to different bioclimatic conditions, i.e. breeding for tolerance to heat and drought stress. A significant decrease in rainfall is predicted for the Mediterranean region, which will lead to more severe drought events, increasing temperatures and UV radiation. As conditions become more extreme, it is important to understand the genetic adaptations of Mediterranean plants, therefore, future studies on Dalmatian pyrethrum should pay great attention to clarifying the latter so that their fate under changing climatic conditions can be predicted and strategies and tools for their protection and sustainable production can be developed.

## Data availability statement

The original contributions presented in the study are included in the article/**Supplementary Material**. Further inquiries can be directed to the corresponding author.

## Author contributions

MG: Conceptualization, Methodology, Writing – original draft, Writing – review & editing, Formal Analysis, Investigation. NJ: Writing – original draft, Writing – review & editing. FV: Conceptualization, Investigation, Methodology, Visualization, Writing – original draft, Writing – review & editing. ZL: Writing – original draft, Writing – review & editing. AT: Writing – original draft, Writing – review & editing. ZS: Conceptualization, Formal Analysis, Methodology, Supervision, Writing – original draft, Writing – review & editing.
