# Supplementary material for: Local adaptation to the native environment affects pyrethrin variability in Dalmatian pyrethrum populations
Source: Front Plant Sci. 2024 Jun 21;15:1404614. doi: 10.3389/fpls.2024.1404614 (PMC11232531; doi:10.3389/fpls.2024.1404614)
Supplement: Supplementary file 1 [file Table_1.pdf]

**Table S1. Bioclimatic variables of 15 Dalmatian pyrethrum sampling sites and bioclimatic groups obtained from the WorldClim database (available at: www.worldclim.org)**

| No. | Bioclimatic variable | Sampling sites (populations) | | | | | | | | | | | | | | |
|---|---|---|---|---|---|---|---|---|---|---|---|---|---|---|---|---|
| | | P01 | P02 | P03 | P04 | P05 | P06 | P07 | P08 | P09 | P10 | P11 | P12 | P13 | P14 | P15 |
| BIO01 | Annual Mean Temperature | 22.25 | 22.12 | 20.01 | 17.31 | 14.83 | 24.17 | 27.98 | 26.66 | 27.00 | 21.02 | 21.97 | 22.82 | 20.86 | 20.07 | 23.56 |
| BIO02 | Mean Diurnal Range (Mean of monthly (max temp - min temp | 10.82 | 11.62 | 12.30 | 13.66 | 13.42 | 10.93 | 12.55 | 12.63 | 13.14 | 12.79 | 14.25 | 13.96 | 13.57 | 13.82 | 13.34 |
| BIO03 | Isothermality (BIO02/BIO07) (* 100) | 50.12 | 56.67 | 58.29 | 60.17 | 58.59 | 53.33 | 56.79 | 54.89 | 55.69 | 53.97 | 55.02 | 54.10 | 53.24 | 53.35 | 52.12 |
| BIO04 | Temperature Seasonality (standard deviation *100) | 369.68 | 298.68 | 290.20 | 294.42 | 315.81 | 342.28 | 356.35 | 383.06 | 384.76 | 413.49 | 436.52 | 449.68 | 453.35 | 456.40 | 470.88 |
| BIO05 | Max Temperature of Warmest Month | 32.00 | 31.40 | 29.60 | 27.60 | 25.20 | 33.60 | 38.70 | 37.70 | 38.50 | 32.30 | 34.40 | 35.10 | 33.10 | 32.60 | 35.70 |
| BIO06 | Min Temperature of Coldest Month | 10.40 | 10.90 | 8.50 | 4.90 | 2.30 | 13.10 | 16.60 | 14.70 | 14.90 | 8.60 | 8.50 | 9.30 | 7.60 | 6.70 | 10.10 |
| BIO07 | Temperature Annual Range (BIO05-BIO06) | 21.60 | 20.50 | 21.10 | 22.70 | 22.90 | 20.50 | 22.10 | 23.00 | 23.60 | 23.70 | 25.90 | 25.80 | 25.50 | 25.90 | 25.60 |
| BIO08 | Mean Temperature of Wettest Quarter | 23.05 | 24.98 | 22.77 | 18.02 | 15.50 | 27.63 | 31.60 | 27.23 | 27.58 | 21.32 | 22.28 | 23.13 | 21.02 | 20.22 | 23.78 |
| BIO09 | Mean Temperature of Driest Quarter | 17.53 | 19.43 | 17.22 | 14.40 | 11.77 | 24.00 | 28.18 | 30.75 | 31.10 | 21.05 | 21.98 | 19.70 | 17.73 | 16.93 | 20.35 |
| BIO10 | Mean Temperature of Warmest Quarter | 26.50 | 25.47 | 23.27 | 20.63 | 18.47 | 28.08 | 32.02 | 31.00 | 31.37 | 25.85 | 27.12 | 28.17 | 26.28 | 25.52 | 29.17 |
| BIO11 | Mean Temperature of Coldest Quarter | 17.53 | 18.32 | 16.27 | 13.52 | 10.80 | 19.93 | 23.42 | 21.73 | 22.05 | 15.87 | 16.48 | 17.18 | 15.25 | 14.42 | 17.70 |
| BIO12 | Annual Precipitation | 249.00 | 220.00 | 275.00 | 339.00 | 369.00 | 117.00 | 68.00 | 103.00 | 116.00 | 238.00 | 219.00 | 218.00 | 256.00 | 264.00 | 229.00 |
| BIO13 | Precipitation of Wettest Month | 44.00 | 48.00 | 58.00 | 68.00 | 75.00 | 25.00 | 16.00 | 16.00 | 17.00 | 50.00 | 50.00 | 47.00 | 49.00 | 50.00 | 43.00 |
| BIO14 | Precipitation of Driest Month | 3.00 | 4.00 | 6.00 | 7.00 | 6.00 | 2.00 | 1.00 | 3.00 | 3.00 | 5.00 | 4.00 | 6.00 | 8.00 | 8.00 | 9.00 |
| BIO15 | Precipitation Seasonality (Coefficient of Variation) | 66.72 | 79.29 | 79.72 | 78.53 | 81.17 | 69.51 | 56.97 | 44.37 | 42.93 | 78.15 | 86.36 | 75.08 | 68.32 | 67.33 | 61.41 |
| BIO16 | Precipitation of Wettest Quarter | 101.00 | 97.00 | 117.00 | 139.00 | 154.00 | 46.00 | 27.00 | 36.00 | 40.00 | 129.00 | 128.00 | 119.00 | 131.00 | 134.00 | 114.00 |
| BIO17 | Precipitation of Driest Quarter | 15.00 | 19.00 | 23.00 | 25.00 | 28.00 | 11.00 | 11.00 | 18.00 | 19.00 | 22.00 | 18.00 | 23.00 | 28.00 | 30.00 | 29.00 |
| BIO18 | Precipitation of Warmest Quarter | 94.00 | 95.00 | 116.00 | 138.00 | 151.00 | 44.00 | 24.00 | 26.00 | 27.00 | 59.00 | 50.00 | 48.00 | 64.00 | 67.00 | 50.00 |
| BIO19 | Precipitation of Coldest Quarter | 15.00 | 23.00 | 28.00 | 30.00 | 35.00 | 18.00 | 15.00 | 23.00 | 27.00 | 28.00 | 23.00 | 27.00 | 30.00 | 31.00 | 32.00 |

| No. | Bioclimatic variable | Boclimatic group | | | | |
|---|---|---|---|---|---|---|
| | | A | B | C | D | E |
| BIO01 | Annual Mean Temperature | 23.21 | 21.06 | 16.07 | 27.22 | 21.72 |
| BIO02 | Mean Diurnal Range (Mean of monthly (max temp - min temp | 10.88 | 11.96 | 13.54 | 12.77 | 13.62 |
| BIO03 | Isothermality (BIO02/BIO07) (* 100) | 51.72 | 57.48 | 59.38 | 55.79 | 53.63 |
| BIO04 | Temperature Seasonality (standard deviation *100) | 355.98 | 294.44 | 305.11 | 374.73 | 446.72 |
| BIO05 | Max Temperature of Warmest Month | 32.8 | 30.5 | 26.4 | 38.3 | 33.87 |
| BIO06 | Min Temperature of Coldest Month | 11.75 | 9.7 | 3.6 | 15.4 | 8.47 |
| BIO07 | Temperature Annual Range (BIO05-BIO06) | 21.05 | 20.8 | 22.8 | 22.9 | 25.4 |
| BIO08 | Mean Temperature of Wettest Quarter | 25.34 | 23.88 | 16.76 | 28.81 | 21.96 |
| BIO09 | Mean Temperature of Driest Quarter | 20.77 | 18.33 | 13.08 | 30.01 | 19.63 |
| BIO10 | Mean Temperature of Warmest Quarter | 27.29 | 24.37 | 19.55 | 31.46 | 27.02 |
| BIO11 | Mean Temperature of Coldest Quarter | 18.73 | 17.29 | 12.16 | 22.4 | 16.15 |
| BIO12 | Annual Precipitation | 183 | 247.5 | 354 | 95.67 | 237.33 |
| BIO13 | Precipitation of Wettest Month | 34.5 | 53 | 71.5 | 16.33 | 48.17 |
| BIO14 | Precipitation of Driest Month | 2.5 | 5 | 6.5 | 2.33 | 6.67 |
| BIO15 | Precipitation Seasonality (Coefficient of Variation) | 68.12 | 79.5 | 79.85 | 48.09 | 72.77 |
| BIO16 | Precipitation of Wettest Quarter | 73.5 | 107 | 146.5 | 34.33 | 125.83 |
| BIO17 | Precipitation of Driest Quarter | 13 | 21 | 26.5 | 16 | 25 |
| BIO18 | Precipitation of Warmest Quarter | 69 | 105.5 | 144.5 | 25.67 | 56.33 |
| BIO19 | Precipitation of Coldest Quarter | 16.5 | 25.5 | 32.5 | 21.67 | 28.5 |
