# Supplementary material for: Local adaptation to the native environment affects pyrethrin variability in Dalmatian pyrethrum populations
Source: Front Plant Sci. 2024 Jun 21;15:1404614. doi: 10.3389/fpls.2024.1404614 (PMC11232531; doi:10.3389/fpls.2024.1404614)
Supplement: Supplementary file 2 [file Table_2.pdf]

Table S2. Total diversity of six pyrethrin compounds (expressed as % of total pyrethrin), total pyrethrin content (% of flower dry weight) and pyrethrin I / pyrethrin II ratio in 15 Dalmatian pyrethrum populations (300 plant samples).

Compound	Average	sd	Range	CV%
Pyrethrin I	48.98	11.30	18.91-76.23	23.07
Pyrethrin II	38.44	11.00	11.80-72.36	28.63
Cinerin I	4.22	1.71	0.87-13.02	40.47
Cinerin II	4.28	2.09	0.33-11.53	48.79
Jasmolin I	2.30	1.03	0.37-6.77	44.94
Jasmolin I	1.79	0.60	0.34-3.74	33.67
Total pyrethrin content	1.01	0.28	0.10-1.94	27.18
Pyrethrin I / Pyrethrin II Ratio	1.52	0.97	0.26-6.46	63.78
