# Supplementary material for: Local adaptation to the native environment affects pyrethrin variability in Dalmatian pyrethrum populations
Source: Front Plant Sci. 2024 Jun 21;15:1404614. doi: 10.3389/fpls.2024.1404614 (PMC11232531; doi:10.3389/fpls.2024.1404614)
Supplement: Supplementary file 3 [file Table_3.pdf]

Table S3. Coefficients of variation (*CV*) and the range of six pyrethrin compounds (expressed as % of total pyrethrin), total pyrethrin content (% of flower dry weight) and pyrethrin I / pyrethrin II ratio in 15 Dalmatian pyrethrum populations and five bioclimatic groups.

	Pyrethrin I		Pyrethrin II		Cinerin I		Cinerin II		Jasmolin I		Jasmolin I		Total pyrethrin content		Pyrethrin I/Pyrethrin II Ratio	
Population ID	CV	Range	CV	Range	CV	Range	CV	Range	CV	Range	CV	Range	CV	Range	CV	Range
P01	19.73	35.94-73.89	27.31	15.31-55.77	30.90	1.21-4.96	50.73	0.89-5.87	34.20	1.67-5.62	32.85	1.10-3.34	21.32	0.65-1.59	58.02	0.65-4.83
P02	17.42	30.34-57.82	14.37	30.87-56.07	30.49	2.03-7.63	31.10	2.71-10.49	33.40	0.81-2.74	17.16	1.22-2.12	19.22	0.54-1.37	38.45	0.54-1.87
P03	19.24	37.77-68.50	26.79	19.08-56.38	37.54	0.87-6.74	44.79	1.43-7.14	33.92	0.99-4.21	24.88	0.95-2.75	24.27	0.33-1.27	53.81	0.67-3.59
P04	22.96	18.91-46.15	17.14	38.66-72.36	42.27	1.24-8.25	27.41	3.97-11.53	33.25	0.37-1.95	18.43	1.33-2.59	25.01	0.50-1.35	36.31	0.26-1.10
P05	24.58	28.96-71.16	24.00	21.51-60.84	46.07	1.39-5.65	50.36	1.09-8.91	33.14	0.73-2.97	35.02	0.84-3.07	37.25	0.22-1.85	65.41	0.49-3.31
P06	25.72	21.00-63.41	24.56	25.49-69.12	38.51	1.14-7.05	34.67	2.30-9.81	30.03	0.92-3.20	31.03	1.01-3.39	32.13	0.10-1.40	54.80	0.30-2.49
P07	20.75	32.75-72.81	26.33	21.99-53.50	36.03	0.87-6.72	48.55	0.33-6.68	24.95	1.31-4.09	37.48	0.93-3.74	36.47	0.61-1.94	47.45	0.61-3.29
P08	18.49	28.94-68.13	28.99	12.03-56.71	51.60	2.68-13.02	33.44	2.42-7.54	27.75	1.57-4.55	31.81	0.98-3.28	29.15	0.66-1.57	72.02	0.51-5.66
P09	12.87	35.32-62.73	15.29	26.96-46.38	30.10	2.71-7.60	39.64	1.78-8.12	23.08	1.17-2.79	34.13	0.84-2.98	17.93	0.60-1.08	28.40	0.76-2.25
P10	23.30	36.58-76.23	29.11	11.80-54.06	28.93	2.41-8.24	44.54	1.74-9.62	32.87	1.02-3.09	37.42	0.34-2.36	15.80	0.80-1.41	85.02	0.68-6.46
P11	20.30	33.59-72.90	29.56	16.07-55.13	32.01	2.16-6.74	44.88	1.71-7.64	35.88	1.32-4.89	41.04	0.62-3.39	16.35	0.72-1.42	58.21	0.61-4.54
P12	12.59	42.26-68.09	24.82	14.23-50.04	30.25	1.88-8.36	29.72	1.83-6.26	45.17	1.32-6.11	24.26	0.65-2.19	20.30	0.61-1.23	48.93	0.86-4.78
P13	16.50	35.59-66.69	20.09	19.33-54.27	33.26	1.84-7.38	33.04	1.58-7.07	33.68	0.90-4.17	36.45	0.87-3.06	29.60	0.40-1.31	45.69	0.69-3.45
P14	13.72	37.57-59.95	19.34	25.95-49.80	27.19	2.56-7.68	26.26	2.53-6.33	24.72	1.42-3.82	23.96	1.27-3.00	12.28	0.89-1.53	33.60	0.75-2.31
P15	7.54	56.41-73.52	23.35	13.95-36.49	46.45	1.04-6.15	50.23	0.58-3.11	20.47	2.98-6.77	21.46	1.18-2.27	19.67	0.81-1.65	30.35	1.55-5.27
Bioclimatic group																
A	24.87	21.00-73.89	27.53	15.31-69.12	38.82	1.14-7.05	50.42	0.89-9.81	40.86	0.92-5.62	31.53	1.01-3.39	29.12	0.10-1.59	62.02	0.30-4.83
B	22.51	30.34-68.50	23.17	19.08-56.38	33.69	0.87-7.63	44.49	1.43-10.49	44.70	0.81-4.21	21.48	0.95-2.75	21.59	0.33-1.37	59.06	0.54-3.59
C	27.81	18.91-71.16	21.17	21.51-72.36	48.92	1.24-8.25	50.76	1.09-11.53	36.76	0.37-2.97	27.35	0.84-3.07	33.12	0.22-1.85	65.00	0.26-3.31
D	17.61	28.94-72.81	23.83	12.03-56.71	43.30	0.87-13.02	40.54	0.33-8.12	29.57	1.17-4.55	37.09	0.84-3.74	33.16	0.60-1.94	53.35	0.51-5.66
E	19.62	33.59-76.23	29.01	11.80-55.13	35.35	1.04-8.36	49.13	0.58-9.62	44.76	0.90-6.77	34.90	0.34-3.39	21.95	0.40-1.65	60.04	0.61-6.46
