# Supplementary material for: Local adaptation to the native environment affects pyrethrin variability in Dalmatian pyrethrum populations
Source: Front Plant Sci. 2024 Jun 21;15:1404614. doi: 10.3389/fpls.2024.1404614 (PMC11232531; doi:10.3389/fpls.2024.1404614)
Supplement: Supplementary file 4 [file Table_4.pdf]

Table S4. Pearson correlation coefficients between 19 bioclimatic variables at 15 Dalmatian pyrethrum sampling sites.

Variable	BIO01	BIO02	BIO03	BIO04	BIO05	BIO06	BIO07	BIO08	BIO09	BIO10	BIO11	BIO12	BIO13	BIO14	BIO15	BIO16	BIO17	BIO18	BIO19
BIO01		ns	ns	ns	***	***	ns	***	***	***	***	***	***	*	**	***	*	***	*
BIO02	-0.268		ns	ns	ns	ns	***	ns	ns	ns	ns	ns	ns	*	ns	ns	*	ns	**
BIO03	-0.357	0.251		**	ns	ns	ns	ns	ns	ns	ns	ns	ns	ns	ns	ns	ns	ns	ns
BIO04	0.246	0.499	-0.692		ns	ns	***	ns	ns	ns	ns	ns	ns	ns	ns	ns	ns	*	ns
BIO05	0.953	0.002	-0.429	0.503		***	ns	***	***	***	***	***	***	ns	**	**	ns	***	ns
BIO06	0.976	-0.433	-0.265	0.045	0.865		ns	***	***	***	***	***	***	**	**	***	**	***	**
BIO07	-0.065	0.843	-0.308	0.876	0.240	-0.278		ns	ns	ns	ns	ns	ns	*	ns	ns	*	ns	ns
BIO08	0.954	-0.434	-0.217	0.012	0.836	0.984	-0.304		***	***	***	***	***	**	*	***	**	***	**
BIO09	0.933	-0.160	-0.171	0.162	0.893	0.918	-0.066	0.870		***	***	***	***	**	**	***	*	***	ns
BIO10	0.976	-0.136	-0.484	0.451	0.990	0.909	0.136	0.882	0.893		***	***	***	ns	**	***	*	***	*
BIO11	0.977	-0.392	-0.219	0.034	0.871	0.997	-0.263	0.983	0.924	0.907		***	***	**	**	***	**	***	**
BIO12	-0.964	0.315	0.268	-0.146	-0.893	-0.967	0.163	-0.961	-0.945	-0.921	-0.964		***	**	**	***	**	***	**
BIO13	-0.967	0.329	0.342	-0.185	-0.903	-0.963	0.136	-0.930	-0.946	-0.931	-0.957	0.978		**	***	***	**	***	*
BIO14	-0.603	0.578	-0.008	0.373	-0.413	-0.711	0.587	-0.703	-0.647	-0.471	-0.704	0.706	0.653		ns	***	***	ns	***
BIO15	-0.750	0.134	0.268	-0.212	-0.737	-0.721	-0.016	-0.635	-0.766	-0.735	-0.722	0.694	0.818	0.311		**	ns	*	ns
BIO16	-0.890	0.475	0.107	0.138	-0.744	-0.949	0.411	-0.932	-0.893	-0.788	-0.949	0.936	0.944	0.774	0.759		***	**	**
BIO17	-0.637	0.644	0.098	0.324	-0.440	-0.739	0.590	-0.753	-0.609	-0.515	-0.731	0.722	0.656	0.953	0.238	0.761		ns	***
BIO18	-0.891	-0.020	0.507	-0.593	-0.961	-0.796	-0.300	-0.773	-0.856	-0.953	-0.790	0.876	0.868	0.385	0.636	0.677	0.420		ns
BIO19	-0.628	0.671	0.299	0.186	-0.453	-0.707	0.502	-0.723	-0.499	-0.537	-0.690	0.662	0.619	0.842	0.231	0.674	0.933	0.415	

*ns - non-significant; * - significant at $P < 0.05$; ** - significant at $P < 0.01$; *** - significant at $P < 0.001$.
