# Supplementary material for: Local adaptation to the native environment affects pyrethrin variability in Dalmatian pyrethrum populations
Source: Front Plant Sci. 2024 Jun 21;15:1404614. doi: 10.3389/fpls.2024.1404614 (PMC11232531; doi:10.3389/fpls.2024.1404614)
Supplement: Supplementary file 5 [file Table_5.pdf]

Table S5. Pearson correlation coefficients between 19 bioclimatic variables and scores of the first three PCs.

Variable	PC1		PC2		PC3	
BIO01	-0.979	***	0.156	ns	0.037	ns
BIO02	0.397	ns	0.710	**	0.441	ns
BIO03	0.330	ns	-0.440	ns	0.798	***
BIO04	-0.122	ns	0.933	***	-0.322	ns
BIO05	-0.890	***	0.439	ns	0.048	ns
BIO06	-0.989	***	-0.054	ns	0.051	ns
BIO07	0.210	ns	0.946	***	-0.006	ns
BIO08	-0.967	***	-0.093	ns	0.050	ns
BIO09	-0.933	***	0.132	ns	0.251	ns
BIO10	-0.929	***	0.352	ns	-0.040	ns
BIO11	-0.984	***	-0.047	ns	0.103	ns
BIO12	0.984	***	-0.041	ns	-0.073	ns
BIO13	0.982	***	-0.094	ns	-0.062	ns
BIO14	0.731	**	0.542	*	0.036	ns
BIO15	0.729	**	-0.258	ns	-0.265	ns
BIO16	0.945	***	0.203	ns	-0.184	ns
BIO17	0.754	**	0.536	*	0.184	ns
BIO18	0.844	***	-0.489	ns	0.054	ns
BIO19	0.724	**	0.446	ns	0.400	ns
Eigenvalue	12.390		3.974		1.331	
% variance	65.209		20.914		7.004	

*ns - non-significant; * - significant at $P < 0.05$; ** - significant at $P < 0.01$; *** - significant at $P < 0.001$.
