# Supplementary material for: Local adaptation to the native environment affects pyrethrin variability in Dalmatian pyrethrum populations
Source: Front Plant Sci. 2024 Jun 21;15:1404614. doi: 10.3389/fpls.2024.1404614 (PMC11232531; doi:10.3389/fpls.2024.1404614)
Supplement: Supplementary file 6 [file Table_6.pdf]

Table S6. Pearson correlation coefficients between six pyrethrin compounds and scores of the first two PCs.

Compound	PC1		PC2	
Pyrethrin I	-0.993	***	0.028	ns
Pyrethrin II	0.971	***	-0.196	***
Cinerin I	-0.231	***	0.867	***
Cinerin II	0.749	***	0.560	***
Jasmolin I	-0.797	***	-0.352	***
Jasmolin I	0.457	***	-0.619	***
Eigenvalue	3.388		1.611	
% variance	56.460		26.846	

*ns - non-significant; * - significant at $P < 0.05$; ** - significant at $P < 0.01$; *** - significant at $P < 0.001$
