# Supplementary material for: Local adaptation to the native environment affects pyrethrin variability in Dalmatian pyrethrum populations
Source: Front Plant Sci. 2024 Jun 21;15:1404614. doi: 10.3389/fpls.2024.1404614 (PMC11232531; doi:10.3389/fpls.2024.1404614)
Supplement: Supplementary file 7 [file Table_7.pdf]

Table S7. Pearson correlation coefficients between 19 bioclimatic variables and six pyrethrin compounds, the total pyrethrin content and the pyrethrin I / pyrethrin II ratio

Variable		Pyrethrin I		Pyrethrin II		Cinerin I		Cinerin II		Jasmolin I		Jasmolin I		Total pyrethrin content		Pyrethrin I / Pyrethrin II Ratio	
Latitude (N)		-0.250	***	0.288	***	-0.262	***	0.068	ns	-0.213	***	0.134	*	-0.079	ns	-0.215	***
Longitude (E)		0.344	***	-0.371	***	0.217	***	-0.168	**	0.315	***	-0.145	*	0.133	*	0.302	***
BIO01	Annual Mean Temperature	0.243	***	-0.277	***	0.163	**	-0.123	*	0.343	***	0.081	ns	0.031	ns	0.189	**
BIO02	Mean Diurnal Range (Mean of monthly (max temp - min temp))	0.105	ns	-0.117	*	0.125	*	-0.019	ns	0.036	ns	-0.183	**	0.041	ns	0.096	ns
BIO03	Isothermality (P2/P7) (* 100)	-0.355	***	0.353	***	-0.036	ns	0.284	***	-0.426	***	-0.063	ns	-0.190	***	-0.290	***
BIO04	Temperature Seasonality (standard deviation *100)	0.412	***	-0.428	***	0.164	**	-0.260	***	0.425	***	-0.085	ns	0.195	***	0.348	***
BIO05	Max Temperature of Warmest Month	0.322	***	-0.359	***	0.208	***	-0.166	**	0.408	***	0.035	ns	0.074	ns	0.255	***
BIO06	Min Temperature of Coldest Month	0.161	**	-0.193	***	0.132	*	-0.071	ns	0.261	***	0.110	ns	-0.002	ns	0.118	*
BIO07	Temperature Annual Range (P5-P6)	0.305	***	-0.315	***	0.142	*	-0.180	**	0.277	***	-0.146	*	0.147	*	0.260	***
BIO08	Mean Temperature of Wettest Quarter	0.153	**	-0.181	**	0.112	ns	-0.075	ns	0.256	***	0.120	*	-0.016	ns	0.110	ns
BIO09	Mean Temperature of Driest Quarter	0.155	**	-0.198	***	0.215	***	-0.028	ns	0.216	***	0.048	ns	-0.004	ns	0.117	*
BIO10	Mean Temperature of Warmest Quarter	0.316	***	-0.351	***	0.186	**	-0.172	**	0.411	***	0.056	ns	0.071	ns	0.252	***
BIO11	Mean Temperature of Coldest Quarter	0.158	**	-0.190	***	0.132	*	-0.067	ns	0.258	***	0.103	ns	-0.012	ns	0.117	*
BIO12	Annual Precipitation	-0.145	*	0.185	**	-0.180	**	0.037	ns	-0.238	***	-0.110	ns	-0.001	ns	-0.102	ns
BIO13	Precipitation of Wettest Month	-0.165	**	0.202	***	-0.159	**	0.069	ns	-0.282	***	-0.133	*	-0.017	ns	-0.117	*
BIO14	Precipitation of Driest Month	0.100	ns	-0.090	ns	-0.047	ns	-0.091	ns	0.071	ns	-0.127	*	0.055	ns	0.126	*
BIO15	Precipitation Seasonality (Coefficient of Variation)	-0.187	**	0.207	***	-0.077	ns	0.143	*	-0.331	***	-0.124	*	-0.049	ns	-0.137	*
BIO16	Precipitation of Wettest Quarter	-0.043	ns	0.072	ns	-0.087	ns	0.007	ns	-0.164	**	-0.164	**	0.048	ns	-0.015	ns
BIO17	Precipitation of Driest Quarter	0.069	ns	-0.052	ns	-0.040	ns	-0.076	ns	0.009	ns	-0.149	**	0.067	ns	0.081	ns
BIO18	Precipitation of Warmest Quarter	-0.299	***	0.345	***	-0.257	***	0.117	*	-0.359	***	-0.020	ns	-0.079	ns	-0.240	***
BIO19	Precipitation of Coldest Quarter	0.009	ns	0.008	ns	-0.018	ns	-0.010	ns	-0.109	ns	-0.211	***	-0.023	ns	0.034	ns

*ns - non-significant; * - significant at $P < 0.05$; ** - significant at $P < 0.01$; *** - significant at $P < 0.001$
