# Supplementary figures and images for: Local adaptation to the native environment affects pyrethrin variability in Dalmatian pyrethrum populations
Source: Front Plant Sci. 2024 Jun 21;15:1404614. doi: 10.3389/fpls.2024.1404614 (PMC11232531; doi:10.3389/fpls.2024.1404614)

**Figure S1. HPLC-UV-DAD chromatogram of Dalmatian pyrethrum extract**

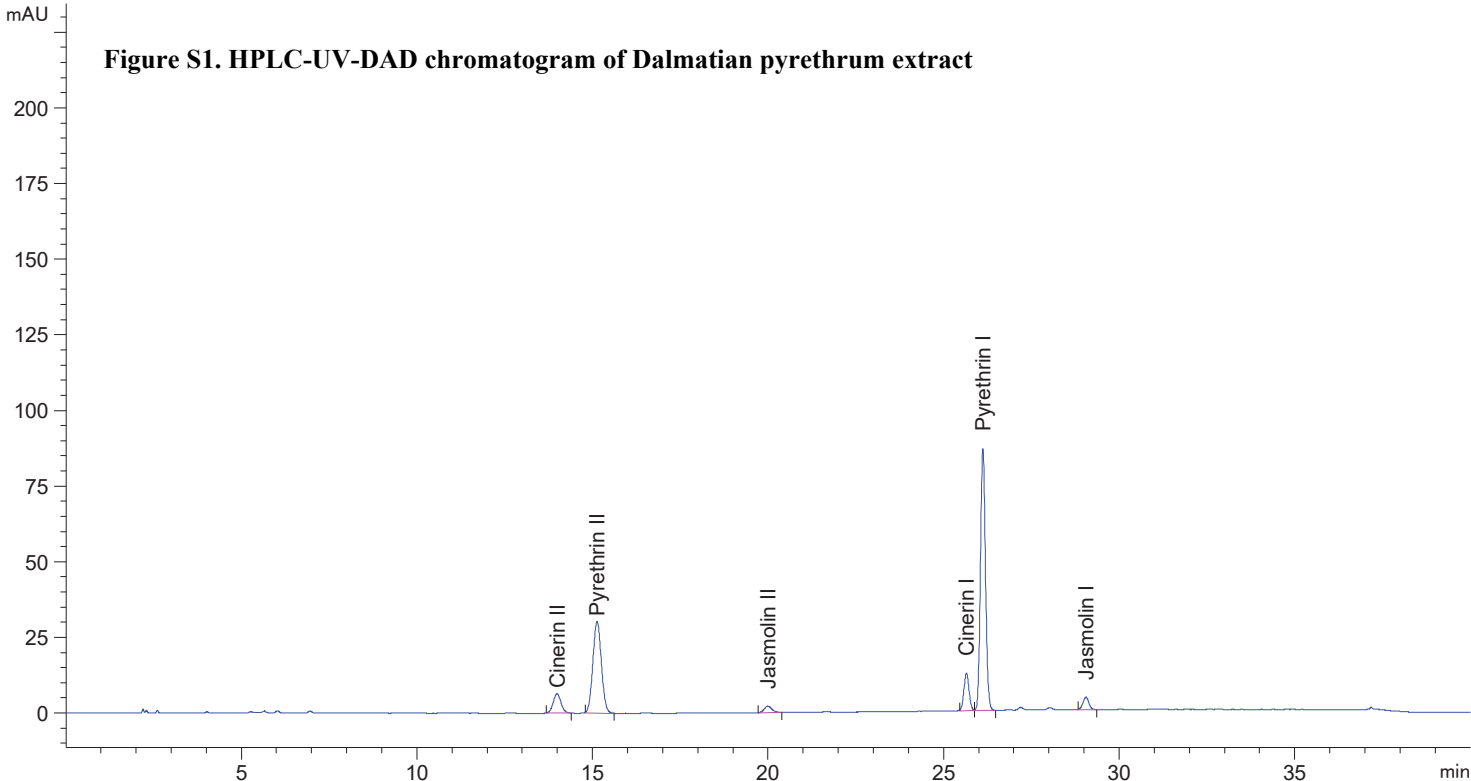

Supplement: Supplementary file 8 [file Image_1.pdf]
